# Bringing science back to the core: A financially sustainable model for core facilities

**DOI:** 10.1038/s44319-023-00032-2

**Published:** 2023-12-20

**Authors:** Juan J Garcia-Vallejo

**Affiliations:** 1 Amsterdam UMC – Location Vrije Universiteit Amsterdam, Molecular Cell Biology & Immunology, De Boelelaan 1117, Amsterdam, The Netherlands; 2 Cancer Center Amsterdam, Biomarkers, Amsterdam, The Netherlands; 3 Amsterdam Infection & Immunity, Cancer Immunology, Amsterdam, The Netherlands

**Keywords:** Economics, Law & Politics, Methods & Resources

## Abstract

A flat-fee user charge by core facilities would greatly benefit both scientists and core facility staff and help them to focus on scientific questions rather than financial considerations.

**Figure:**
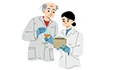

Core facilities, shared research resources, or shared resource laboratories (SRLs), represent a key asset for research institutions. The increasing price of cutting-edge technologies makes it no longer possible for individual research groups or departments to be able to afford high-end instrumentation on their own. Instead, research organizations use SRLs to lower operational costs and to concentrate expertise by centralizing expensive and sophisticated highly specialized instrumentation along with skilled staff to maintain the instruments and help users with training, operation, and data analysis (Farber and Weiss, 2011; Gould, 2015).

In addition to these critical functions, SRLs play a key role in providing quality assurance and are often seen by researchers across the organization as collaborators rather than service providers. Most importantly and beyond the operational and financial motivations of centralizing expertise and equipment, SRLs are essential for research organizations in catalyzing competitive high-quality research.

Most importantly […] SRLs are essential for research organizations in catalyzing competitive high-quality research.

## Cost accounting for SRL usage

With the establishment of SRLs comes the need for spreading the costs of the centralized instrumentation and personnel across the research departments that make use of their services. The goal is to keep the costs for users lower than the cost of implementing the technology in their own department or laboratory. In addition, SRLs should spread costs across users in a fair and appropriate manner while achieving a neutral financial balance every fiscal year.

Traditionally, SRLs have been using time-driven activity-based costing (ABC) (Kaplan and Anderson, 2004) to calculate instrument and service usage fees. In this model, an hourly fee is calculated by summing up the individual contribution of the instrument service contracts and direct costs, such as electricity, buffers, medical gasses or calibration beads, and the operator time involved in maintaining instruments, customer service, and consultation. Some organizations might also add the instrument depreciation to this formula.

The collection of usage fees helps to maintain the operation of the core facility, but relying on fees alone can be problematic. For example, SRLs supported by internal funding may be able to offer lower usage fees and thereby attract customers from external organizations in which SRLs are less subsidized. Another disadvantage is the difficulties in predicting the costs involved in a research project ahead of time with the result that the accumulated total costs might be higher than the funding allows. Researchers may end up making decisions on whether to perform an experiment or not based on financial rather than scientific reasons.

Researchers may end up making decisions on whether to perform an experiment or not based on financial rather than scientific reasons.

**Figure 1:**
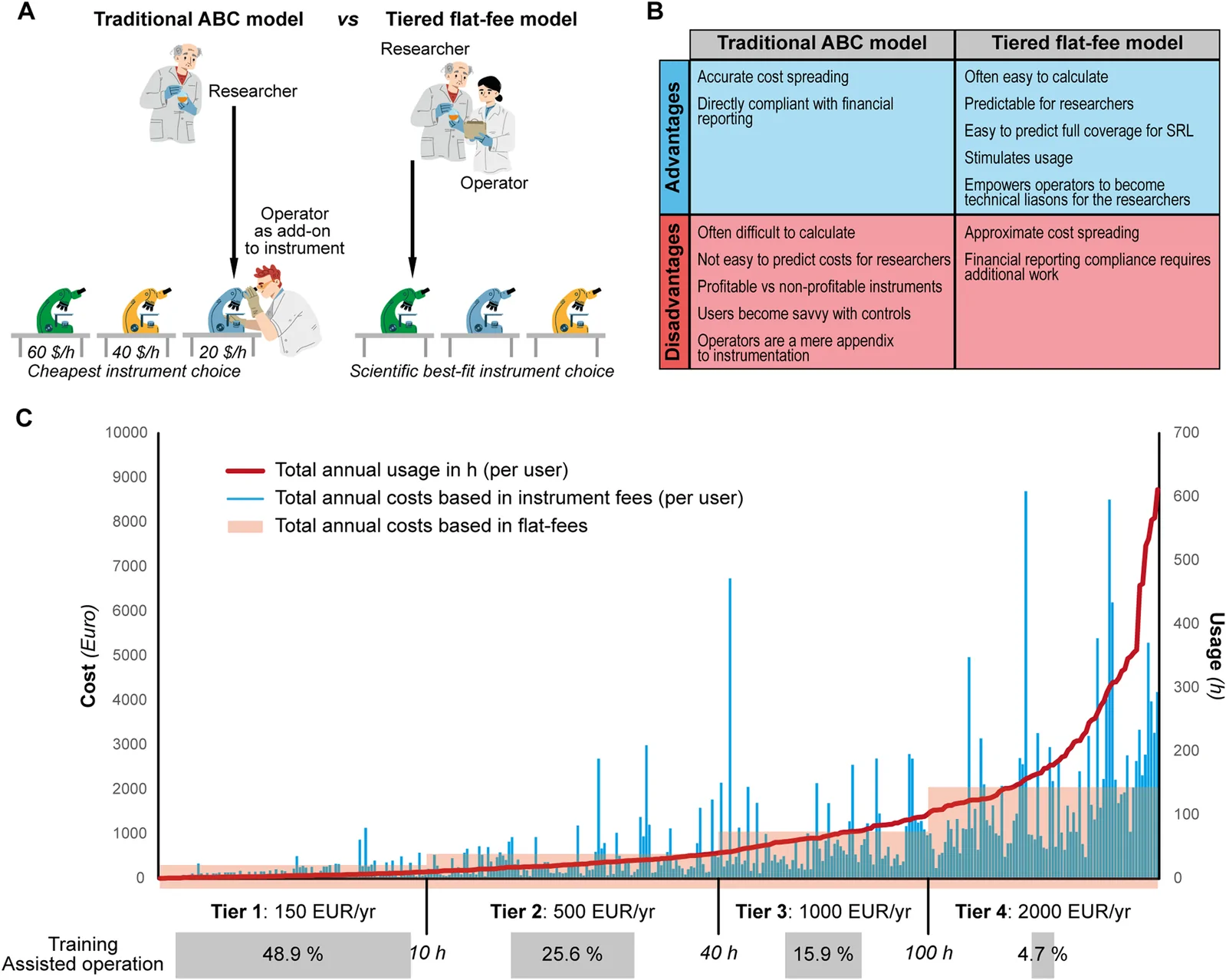
Side-by-side comparison of a traditionally calculated usage fee (Traditional ABC model) vs a tiered flat-fee model as presented in this article. (**A**) In a traditional ABC model, the researcher picks and chooses resources from the SRL and the operator can be seen as a mere administrator or add on to the instrumentation, whereas in the tiered flat-fee model, the operator collaborates with the researcher in selecting the most appropriate technology for the required scientific question. (**B**) Summary of the advantages and disadvantages of both models. (**C**) Comparison of both models based on usage data from our Microscopy and Cytometry Core Facility at Amsterdam UMC. The left *Y* axis indicates costs (in Euro) and is linked to the blue bars (total annual cost per user based on instrument usage fees) and the orange box (total annual cost per usage tier based on a flat-fee model), the right *Y* axis indicates usage (in hours) and is linked to the red line (total annual usage time). The *X* axis displays all individual users of the core facility ranked by total usage time. The gray bar underneath the *X* axis indicates the percentage of operator-assisted usage time per tier. Tiers are indicated in the X axis (less than 10 h/yr, between 10 and 40 h/yr, between 40 and 100 h/yr, and more than 100 h/yr).

Furthermore, when multiple instruments are available to perform an experiment, scientists might be tempted to use the instrument with lower usage fees, even if this instrument represents a suboptimal solution (Fig. 1A). Or worse, researchers might be tempted to skip important controls to save money. Finally, the scientific and technical expertise present in the SRL is often ignored when the users make financially-driven choices, which ultimately demotivates SRL personnel, as they might end up seeing themselves merely as extensions of an instrument, just administrators of equipment, rather than true scientific and technical liaisons for researchers (Fig. 1B).

## A tiered flat-fee model

To address these problems, I propose an alternative financial model, based on a tiered flat-fee system, that would have multiple advantages. First, instrument choice is no longer dependent on instrument-linked usage rates since all instruments are rated equally. This brings back the focus on scientific arguments rather than financial ones and places the role of the SRL operators at the center of the decision-making which is critical for the success of the experiment. Second, a tiered flat-fee model makes expenses easily predictable which helps scientists to make decisions early on regarding the volume of experimentation needed for the project. Moreover, SRL personnel can make more accurate predictions about the expected yearly income of the SRL, which liberates them from financial pressure and gives them more time to think about what matters, namely science. Third, a tiered flat-fee model stimulates users to perform more experiments, as the rates become cheaper with increasing usage.

… a tiered flat-fee model stimulates users to perform more experiments, as the rates become cheaper with increasing usage.

A tiered flat fee with no differences in price per instrument helps to focus on the SRL as a service driven by capable operators and not a collection of instruments to choose from. This model also means that financial pressures do not drive SRLs to only invest in “workhorses.” On the other side, frequent users are safe from incurring large expenses as they are encouraged to work independently (unassisted), whereas less frequent or less experienced users do not have to pay extra for assistance and training. Finally, the tiered aspect of the system ensures that the costs match a certain usage level, which will be likely perceived as being fair by SRL users.

To demonstrate the value of a tiered flat-fee system, I compared the impact of a usage fee based on activity-based costing (“traditional ABC model”) versus a tiered flat-fee (“tiered flat-fee model”) based on the usage data generated at the Microscopy and Cytometry Core Facility at the Amsterdam UMC (Location VUmc) between January and December 2021. Figure 1C demonstrates that time-driven activity-based instrument usage fees lead to at least one third of the users paying higher annual costs compared to a flat-fee model; and operator-assisted instrument usage is more frequent at the lower usage tiers and decreases as usage increases, suggesting that operator-independent usage is more cost-effective for the facility. Importantly, the total core facility income is comparable between the usage fee-based model (271,000 Euro) and the tiered flat-fee model (288,000 Euro).

Besides the obvious advantage for both users and SRL managers being able to predict their total costs and income, respectively, the implementation of a tiered flat-fee system would have important psychological effects on how both users and operators perceive their roles in the SRL. Facility operators do not have to discuss finances with users and can focus on the needed instrumentation for each individual project. Users, on the other hand, will perceive how progressing to a higher tier may be more cost-effective (Gourville and Soman, 2002), which will stimulate SRL usage. In fact, it is likely that the number of users in tier 4 will increase in time. This should actually be a desired outcome for the organization. This model stimulates usage of internal research infrastructure and, therefore, contributes to improving the return on investment and maximizing the impact on science of the SRLs. In fact, having more users progress to tier 4 will increase SRL revenue and close the gap on financial cost recovery for the organization.

This model stimulates usage of internal research infrastructure and, therefore, contributes to improving the return on investment and maximizing the impact on science of the SRLs.

## One size fits all?

This model is most appropriate for SRLs for which direct variable costs are minimal, as is the case of microscopy and cytometry core facilities, and to some extent for proteomics or metabolomics facilities. For these types of SRLs, the main cost driver are fixed costs, such as personnel, service contracts, and depreciation of instruments; it is therefore easier to achieve a neutral financial balance while offering services at the lowest cost possible for the users. The model also allows for growth as part of the financial target as SRLs may decide based on the expected level of usage whether the financial targets allow the purchase of new instrumentation and/or hiring of additional personnel. Alternatively, the growth of the SRL in response to the need for new technology and expertise could be left out of this billing model and should continue to be covered by the host organization. This aspect is in line with the strategic role of SRLs, which should be used to enable primary processes that lead to the fulfillment of the organization’s leadership vision. Importantly, the model can easily be adapted to increases in expected income targets, since small variations in pricing can easily result in a 20–30% increase in revenue which can effortlessly be anticipated based on usage information.

Reporting to funding agencies is an important administrative task for many SRLs. As some agencies require detailed invoices, it is important that SRLs willing to adopt a tiered flat-fee system need to consider the compatibility of the model with the reporting needs of each funding agency. While some agencies might immediately accept a tiered flat-fee model, others might still demand invoices listing individual instrument fees and the exact number of hours spent. In this event, SRLs can still provide full pricing information based on traditional time-driven activity-based costing and then apply a subsidy to fit the final costs to their tiered flat-fee system. In this setting, the billing is done according to the conventional method and the tiered flat fee is used by the SRLs to subsidize their users, which is generally accepted by funding agencies.

In conclusion, a tiered flat-fee model offers multiple benefits to research organizations in comparison to the classical extended instrument usage fee model. More notably, it helps the organization focus on the primary process, which is to produce great science, and takes away the financial and administrative burden that is asphyxiating our organizations.

## Supplementary information

Peer Review File
