# Supplementary material for: Bringing science back to the core: A financially sustainable model for core facilities
Source: EMBO Rep. 2023 Dec 20;25(2):461–3. doi: 10.1038/s44319-023-00032-2 (PMC10897154; doi:10.1038/s44319-023-00032-2)
Supplement: Supplementary file 1 — Peer Review File [file 44319_2023_32_MOESM1_ESM.pdf]

EMBO reports

Bringing Science back to the Core

Juan Garcia-Vallejo
DOI: 10.15252/embr.202358402

Corresponding author(s): Juan Garcia-Vallejo (jj.garciavallejo@amsterdamumc.nl)

Review Timeline:

Submission Date:	30th Oct 23
Editorial Decision:	15th Nov 23
Revision Received:	17th Nov 23
Accepted:	6th Dec 23

Editor: Holger Breithaupt

Transaction Report: This article was editorially reviewed.
